# Objetos narrativos de las ciencias: de la industrialización a la cobotización de la experiencia interpretativa

**DOI:** 10.18294/sc.2025.5658

**Published:** 2025-08-27

**Authors:** Viviana Martinovich

**Affiliations:** 1 Doctora en Salud Colectiva. Docente-investigadora, Instituto de Salud Colectiva, Universidad Nacional de Lanús, Buenos Aires, Argentina. vivianamartinovich@gmaol.com Buenos Aires Argentina vivianamartinovich@gmaol.com

**Keywords:** Teoría Crítica, Investigación Científica, Industrialización, Cobotización, Critical Theory, Scientific Research, Industrialization, Cobotization

## Abstract

Desde la teoría crítica y la hermenéutica filosófica, en este ensayo se examinan los procesos de industrialización y “cobotización” de la práctica científica, entendida como la incorporación de inteligencia artificial generativa a la producción y validación del conocimiento científico. El texto aborda, en primer lugar, los objetos narrativos de las ciencias, entendidos como dispositivos de formación de consensos al interior de las comunidades científicas; en segundo lugar, los efectos de la industrialización de la práctica científica, la expansión del complejo industrial científico-editorial y la pérdida del sentido social del texto; y, en tercer lugar, el pasaje de la industrialización a la cobotización tanto de la experiencia interpretativa de las ciencias como de los procesos de validación científica que tecnifican la acción comunicativa. Estos procesos, que erosionan y desarticulan las comunidades de lenguaje, ponen de manifiesto la necesidad de recuperar la experiencia interpretativa como una acción intrínsecamente humana, y revalorizar nuestros propios entornos de validación científica para restituir el carácter relacional, situado y reflexivo de la acción comunicativa propios de los objetos narrativos de las ciencias.

## Abstract (en)

Drawing on critical theory and philosophical hermeneutics, this essay examines the processes of industrialization and “cobotization” of scientific practice, understood as the incorporation of generative artificial intelligence into the production and validation of scientific knowledge. The text addresses, first, the narrative objects of science, understood as devices for building consensus within scientific communities; second, the effects of the industrialization of scientific practice, the expansion of the scientific-editorial industrial complex, and the loss of the social meaning of the text; and third, the shift from industrialization to cobotization, both in the interpretive experience of science and in the processes of scientific validation that technify communicative action. These processes, which erode and disarticulate language communities, highlight the need to recover the interpretive experience as an intrinsically human action and to revalue our own environments of scientific validation, in order to restore the relational, situated, and reflective character of communicative action that is intrinsic to the narrative objects of science.

## INTRODUCCIÓN

En este ensayo, retomamos la tradición de la teoría crítica y, en particular, el concepto de “tecnificación del mundo de la vida” de Jürgen Habermas^1^, para analizar uno de los recientes procesos de tecnificación de la acción comunicativa, relacionados con la generalización del uso de la inteligencia artificial generativa en la práctica científica. Nos referimos al vínculo entre seres humanos y bots “colaborativos” o cobots con capacidad de generar interpretaciones científicas del entorno y de las teorías precedentes y traducirlas a lenguajes narrativos que, si bien tienen múltiples limitaciones^2^^,^^3^^,^^4^^,^^5^^,^^6^^,^^7^^,^^8^^,^^9^^,^^10^, aun así, han comenzado a ser considerados válidos por el componente humano de esa relación. Esto ha llevado a un uso compulsivo y acrítico de la inteligencia artificial que, desde ciertos ámbitos, es percibido como una ruptura de las “reglas de juego” y un quiebre del “contrato social” acordado implícitamente al interior de las comunidades científicas^11^^,^^12^. ¿Cuáles son esas reglas de juego que se desarticulan en el proceso de cobotización de la experiencia interpretativa de las ciencias?, ¿qué tipo de acuerdos se resquebrajan?, ¿se trata de un cambio sin precedentes o tiene sus raíces en procesos anteriores?

Para intentar responder a estas preguntas, retomamos los conceptos de la hermenéutica filosófica de Hans-Georg Gadamer y Paul Ricoeur, y la teoría crítica de Herbert Marcuse y Jürgen Habermas, para analizar, en primer lugar, los *objetos narrativos de las ciencias*^13^, entendidos como dispositivos que nuclean conversaciones públicas de las ciencias en el plano narrativo, en los que se producen procesos lingüísticos de formación de consensos al interior de las comunidades científicas. En segundo lugar, se retoman los efectos de la industrialización en la práctica científica, entre ellos, la desarticulación de las complejas y múltiples prácticas relacionales humanas a través de las cuales las comunidades científicas solían consolidar acuerdos, desacuerdos y consensos que admitían la coexistencia de diversas formas humanas de comprender el entorno natural y social. Y, en tercer lugar, se aborda el pasaje de esa industrialización a la *cobotización* tanto de la *experiencia interpretativa de las ciencias* como de los procesos de *validación científica* de esas interpretaciones posibles del entorno, para discutir qué racionalidades se expresan en esas articulaciones. 

## Objetos narrativos de las ciencias

Desde el nacimiento de las ciencias modernas, el texto ha sido el principal dispositivo sobre el que ha girado la reflexión y discusión colectiva de las ciencias. Más allá de las distinciones disciplinarias, en todas las ciencias, los lenguajes humanos fijados en la escritura han sido el medio en el que se expresaban acuerdos, desacuerdos, tensiones y rupturas, sobre la base de ciertas matrices interpretativas con características más reflexivas o más instrumentales, más creativas o más descriptivas, que fueron actuando a lo largo de la historia como hilos conductores de diversas tradiciones científicas^14^^,^^15^^,^^16^.

Sin embargo, esos textos solo se tornaban escenarios de la acción colectiva en la medida que dejaban de ser manuscritos inéditos, limitados a la esfera privada, y se abrían a la posibilidad de atravesar múltiples acciones mediadoras para ingresar a la esfera pública. Es decir, se abrían a la posibilidad de confrontar sus premisas y permitir incluso que el texto sea moldeado sobre la base de las nociones consensuadas por las diversas comunidades científicas en cada momento histórico. Así, el texto pasaba a adoptar la racionalidad y la materialidad propuesta por el dispositivo de publicación elegido, que nucleaba en torno de sí a aquellas personas que compartían algo más que el saber disciplinario. Tal como señalamos en otro texto:

“Las comunidades interpretativas que se conforman en torno a cada revista científica comparten formas comunes de ‘comprenderse’ ante esos textos que cada revista pone en circulación, pero esa forma de comprenderse ante el texto trasciende la disciplina: no es el recorte diciplinar ni temático lo que logra conformar una comunidad interpretativa en torno a una revista científica, sino que es una *interpretación común del mundo*, y ese acuerdo común excede el ámbito de las ciencias y se expande a los intereses del mundo de la vida”.^17^

Estos dispositivos, entendidos como *objetos narrativos de las ciencias*, fueron históricamente los responsables de motorizar los procesos lingüísticos de formación de consensos al interior de las diversas comunidades científicas, lo que demandaba la presencia activa de comunidades de lenguaje que se nuclearan en torno a un “concepto abstracto de mundo”, tal como señala Habermas:

“El mundo sólo cobra objetividad por el hecho de *ser reconocido y considerado* como uno y el mismo mundo *por* una comunidad de sujetos capaces de lenguaje y de acción. El concepto abstracto de mundo es condición necesaria para que los sujetos que actúan comunicativamente puedan entenderse entre sí sobre lo que sucede en el mundo o lo que hay que producir en el mundo. Con esta *práctica comunicativa* se aseguran a la vez del contexto común de sus vidas, del *mundo de la vida* que intersubjetivamente comparten. Este viene delimitado por la totalidad de las interpretaciones que son presupuestas por los participantes como un saber de fondo”.^1^ (cursivas del original)

Esas nociones intersubjetivamente compartidas se ponían en acción tanto en el acto de validar y poner en circulación esos acuerdos a través de estos objetos narrativos, como en el acto de nuclearse y de “comprenderse”^16^ colectivamente ante esos textos. Así, en las primeras décadas del siglo XX, revistas como *Zeitschrift für Sozialforschung*(Revista de Investigación Social) editada por el Instituto de Investigaciones Sociales de Frankfurt y dirigida por Max Horkheimer, nucleaban en torno de sí racionalidades más reflexivas que ponían en cuestionamiento las nociones de teoría y de conocimiento científico, ya no como esferas independientes o “supra-sociales”, sino como esferas fuertemente integradas al aparato social. El conocido editorial de Horkheimer titulado “*Teoría tradicional y crítica*”^18^, seguido por los textos de Horkheimer y Marcuse “*Filosofía y teoría crítica*”^19^ ponían en circulación formas de comprender la “génesis social” de los problemas, entre los que incluían los usos industriales del conocimiento científico^19^. Quienes discutían y otorgaban validez a las expresiones simbólicas que la Escuela de Frankfurt expresaba en sus textos, remitían a un saber de fondo compartido intersubjetivamente por la comunidad de comunicación que se comprendía a sí misma en torno a esos textos.

Del mismo modo que las racionalidades reflexivas y críticas creaban sus escenarios de legitimación, por aquellos años, las racionalidades técnicas transformaban las revistas en el escenario de validación científica y social de los productos resultantes de la investigación industrial. Revistas como *Industrial & Engineering Chemistry*, también nucleaban en torno de sí comunidades que formalizaban consensos lingüísticos, pero asociados a otras racionalidades. En el período de entreguerras, esta revista solía centrarse en los grandes aportes de la industria química a la competitividad militar, oponiéndose a todos los intentos de prohibir el uso de gases venenosos o desfinanciar su investigación. En 1926, en un texto editorial titulado “*The Poison Gas Protocol*” podía leerse:

“Ningún arma de guerra es humanitaria o no sería un arma de guerra, pero en comparación con otras armas, el gas venenoso es humanitario. […] Por una razón muy práctica, los Estados Unidos no deberían aceptar abandonar el gas venenoso. Los Estados Unidos son ricos en recursos químicos, en químicos, en industrias químicas. Están mejor equipados que otras naciones para producir grandes cantidades de gas venenoso en caso de guerra. El gas venenoso posee esta ventaja sobre otras armas: puede experimentarse en secreto. Esta misma característica lo convierte en el arma menos susceptible de regulación mediante tratados. No se puede vigilar todos los tubos de ensayo y contenedores de gas en todos los laboratorios de un país signatario…”.^20^

En 1931, en esta misma revista se validaban los beneficios de la patente del gas venenoso o “*poison gas*” como un “destructor sumamente eficaz de gorgojos y otros insectos que infestan los productos alimenticios almacenados”^21^, sin desconocer su toxicidad para los humanos. 

Y quienes se nucleaban en torno de estas revistas compartían acuerdos que trascendían la disciplina: no era el recorte disciplinario ni temático lo que lograba conformar comunidades de lenguaje en torno a esos objetos narrativos de las ciencias, sino que eran formas de concebir la vida, las relaciones sociales, el sentido de la humanidad, entre muchas otras nociones del mundo de la vida, que trascendían la disciplina. La química no era lo que nucleaba a ciertas comunidades científicas en torno a la validación de agentes químicos de alta toxicidad para fines bélicos o para la producción agrícola, como tampoco era la filosofía la que nucleaba a otras comunidades en torno a los textos de la Escuela de Frankfurt, sino que las diversas comunidades de lenguaje se nucleaban en torno a un “concepto abstracto de mundo” compartido, es decir, se nucleaban en torno a una racionalidad compartida. 

En este sentido, la racionalidad sería esa interfaz entre los saberes precientíficos mediados por los intereses del mundo de la vida, que se organizan y estructuran en formas validadas y acordadas colectivamente de interpretar científicamente el mundo, de tal manera que habrá comunidades que operen racionalidades más reflexivas y críticas y comunidades que operen racionalidades más técnicas e instrumentales, de forma independiente de la disciplina desde la cual se procure comprender un fragmento del entorno, y ese concepto abstracto de mundo compartido proviene del mundo precientífico, es decir, se va a estructurar como un saber científico, pero con componentes de su mundo vital primario. En diálogo con Husserl, Habermas señala que

“…el saber del mundo, aparentemente objetivo de los hechos, está trascendentalmente basado en el mundo precientífico. Los posibles objetos del análisis científico se constituyen de antemano en las autocomprensiones de nuestro mundo vital primario”.^22^

## La industrialización de la práctica científica

Si bien la tensión respecto de quién valida o legitima el conocimiento científico producido estuvo presente en las distintas comunidades científicas desde el origen de las ciencias modernas, las diversas racionalidades, entre ellas, las racionalidades más reflexivas y críticas solían valorar y reivindicar sus propios objetos narrativos porque eran la arena de discusión en la que se moldeaban los acuerdos sobre las formas de interpretar el entorno natural y social, y los procesos de revisión por pares a escala humana hacían las veces de mecanismos de autorregulación que tensionaban las propias nociones de cientificidad^23^^,^^24^. Sin embargo, la masificación de la investigación industrial a gran escala dio paso a un proceso que Habermas denomina como “cientifización de la técnica”:

“Siempre se ha registrado en el capitalismo una presión institucional a elevar la productividad del trabajo por medio de la introducción de nuevas técnicas. Pero las innovaciones dependían de inventos esporádicos que, por su parte, podían ciertamente estar inducidos económicamente, pero que no tenían un carácter organizado. Pero esto ha variado en la medida en que el progreso científico y el progreso técnico han quedado asociados y se alimentan mutuamente. Con la investigación industrial a gran escala, la ciencia, la técnica y la revalorización del capital confluyen en un único sistema”.^22^

Jerome Ravetz en su libro *Scientific knowledge and its social problems*^25^ ubica el inicio del proceso de industrialización de las ciencias en el período de entreguerras y lo asocia con un desplazamiento de la ciencia académica, el predominio de la investigación de capital intensivo y la consiguiente concentración del poder de decisión de la industria sobre la investigación científica, que “introduce en la ciencia la inestabilidad y la sensación de cambio rápido pero descontrolado, característico del mundo de la industria y el comercio”^25^. Sin embargo, al tornarse la ciencia una fuerza productiva al servicio de la industria, uno de los cambios más profundos fue la desarticulación de las redes informales y personales de las comunidades que generaban consensos y acuerdos, para pasar a ser trabajadores asalariados de la industria que se disocian de los efectos sociales de aquello que producen, adoptando una racionalidad técnica. 

En este sentido, la masificación de la investigación industrial a gran escala, asociada a la revalorización y concentración del capital, supuso una expansión del complejo industrial científico-editorial en la segunda mitad del siglo XX^26^, que no solo tecnificó, sino que mercantilizó el texto. 

Ya en 1961, el físico nuclear Alvin Weinberg, al analizar el impacto de la investigación industrial a gran escala se preguntaba: “¿la *Big Science* está arruinando la ciencia?”. Al describir el nuevo engranaje generado por la gran expansión de la investigación industrial en la primera mitad del siglo XX, para inicios de la segunda mitad del siglo ya observaba una “enorme proliferación de escritura científica que, en gran parte, permanece sin leer”^27^. Sin embargo, a pesar de estas voces que alertaban tempranamente sobre los efectos de la industrialización del diálogo científico, las comunidades de distintas regiones del mundo, tanto de países enriquecidos como de países empobrecidos, transferían acríticamente sus entornos de validación y de legitimación a las corporaciones, deslegitimando sus propios escenarios de generación de consensos. Y desde un gran abanico de racionalidades científicas abrazaron durante décadas la industrialización y la mercantilización del texto que otrora fuera la arena de reflexión y de discusión colectiva. 

Cuando esa racionalidad técnica, propia de la investigación industrial, se expandió a otros escenarios de investigación, la presión del capitalismo de elevar la productividad se trasladó a la producción de textos académicos, lo que llevó a una demanda de hiperpublicación de artículos científicos^28^^,^^29^, que vació al texto de su acción comunicativa, desarticulando las comunidades de lenguaje. En este nuevo escenario, esa expansión de la racionalidad técnica por fuera de la industria comienza a provocar un quiebre entre lo que se investiga y el valor o el sentido social de eso que se investiga. De allí que ya no fuera necesario que las y los trabajadores de las ciencias que compartían un mismo territorio llegaran a acuerdos sobre cómo interpretar ese territorio compartido y generaran acciones que permitieran mejorar las condiciones de vida en ese territorio, prácticas que eran comunes en las ciencias del siglo XIX hasta las primeras décadas del siglo XX^17^^,^^30^. ¿Y por qué ya no era necesario? Porque a la par de la ruptura entre el entorno y el texto producido, se produce otro quiebre entre ese texto desterritorializado y la validación científica de ese texto: ya no se requería conformar acuerdos sobre qué se consideraba científicamente válido y qué no en las propias comunidades de lenguaje, porque esa decisión se traslada a los grandes monopolios industriales científico-editoriales. 

Este proceso dio origen a la concentración de los escenarios de validación con la consiguiente expansión del complejo industrial científico-editorial que, al igual que otros procesos industriales, produjo efectos contaminantes de los ecosistemas científicos: se consolidaron procesos de estratificación de las ciencias a través de indicadores de citación^31^, que generaron modelos jerárquicos y competitivos^32^^,^^33^^,^^34^^,^^35^, que desarticularon instancias colectivas y colaborativas de generación de conocimiento. La mercantilización del texto, es decir, la obtención de algún beneficio económico a partir de la publicación de un artículo en revistas industrializadas (sea como pago directo o de forma indirecta a través de otros beneficios) tuvo grandes consecuencias en las diversas racionalidades científicas. 

Para el segmento asociado a la investigación industrial, el proceso de industrialización y de mercantilización del propio texto, que se da en la segunda mitad del siglo XX, fue funcional a los intereses de ciertas industrias. Para que ciertos productos o sustancias industriales puedan ingresar al mercado, se comenzó a exigir la validación científica de sus efectos a través de la realización de ensayos controlados aleatorizados^36^, cuyos resultados debían traducirse a un artículo científico; y solo en el caso de que ese artículo fuera aceptado y publicado en una revista científica se abrían las puertas de la comercialización. De allí que, el texto de ese artículo pasó a ser una moneda de cambio para la inyección de productos o sustancias industriales en el mercado y los altos intereses económicos depositados en la acción de hacer público el texto supuso, entre otras cosas, un aumento del fraude y de las retractaciones^37^^,^^38^^,^^39^^,^^40^^,^^41^. En estos entornos es donde se produjo un aumento de artículos con datos falsos, alteración de imágenes, e incluso la proliferación de las “*paper mills*” (fábricas de artículos) que brindan una variedad de servicios que van desde proporcionar datos de investigación fraudulentos o inventados, gestión de la venta de autorías, hasta la venta de artículos ficticios^42^^,^^43^. En este entorno es que surge la figura editorial de la “retractación”, figura funcional al complejo editorial científico-industrial, dado que, por un lado, la industria editorial se asegura el cobro de los costos de procesamiento del artículo o *article processing charges* (APC) para, luego del cobro, hacer público el fraude y, por otro, esta misma figura ha sido utilizada para retirar de circulación artículos no favorables a los intereses industriales, como el difundido caso que se dio en llamar “*Séralini affair*”^44^^,^^45^.

Pero las ondas expansivas de ese proceso de industrialización y mercantilización del texto alcanzaron también a las racionalidades más reflexivas, y las consecuencias nocivas no se vinculan, en este caso, al fraude o a la figura de la retractación, sino que son mucho más profundas y se relacionan con la pérdida del sentido otorgado a la comprensión colectiva del entorno natural y social, y al texto como el objeto narrativo que traduce esa comprensión. Al instrumentalizarse el texto y disociarse de los problemas del entorno, se produce una desarticulación de aquello que Ricoeur denomina “referencia”, es decir, “su pretensión de alcanzar la realidad”^16^. A diferencia de la literatura de ficción o de la poesía, que admite anular la referencia a una realidad dada, la narrativa científica demanda reponer “el *aquí* y el *ahora*, determinados por la situación del discurso”^16^. 

Para las racionalidades más reflexivas, el texto sería la traducción narrativa de una interpretación posible del entorno, que se moldea, se renueva y se actualiza en las nuevas interpretaciones que colectivamente se realicen de ese texto. Tal como señala Ricoeur, “lo dado a interpretar en un texto es una *proposición de mundo*”^16^, que en las narrativas científicas se configura al momento de ajustar la interpretación del entorno a los acuerdos narrativos que esa racionalidad haya alcanzado. Pero al desarticularse las comunidades de lenguaje y transferir los escenarios de validación a las corporaciones, las racionalidades más reflexivas o críticas terminan adoptando mecanismos interpretativos de otras racionalidades, y los textos adoptan un *aquí-ahora* negado, es decir, un “no lugar” que, en términos de Mac Augé es ese espacio “que no puede definirse ni como espacio de identidad ni como relacional ni como histórico”^46^, resquebrajándose las condiciones para desencadenar procesos colectivos, asociativos, basados en la discusión y la creación de consensos.

## De la industrialización a la cobotización de la experiencia interpretativa

Cuando aún no habíamos desentrañado la magnitud de los efectos que la industrialización produjo en la formación de consensos en el plano narrativo al interior de las racionalidades más reflexivas y en la propia noción de “comunidad de lenguaje”, se generaliza la incorporación de la inteligencia artificial generativa, es decir, bots con capacidad de generar interpretaciones tanto del entorno social y natural como de las tradiciones científicas y traducirlos a un lenguaje y una estructura narrativa válida para los humanos.

La “cobotización” en la industria surgió con la implantación masiva de robots colaborativos (cobots) bajo un modelo productivo basaba en la interacción de personas y máquinas^47^^,^^48^. Sin embargo, actualmente la “cobotización” amplió su interacción e incorporó el paradigma de la “inteligencia aumentada” o “inteligencia híbrida”, referida a la inteligencia artificial generativa potenciada en la interacción con la inteligencia humana^49^^,^^50^. Esta nueva “cobotización” supone la colaboración humana con un agente que puede identificar el estado de un entorno e interpretarlo, para lo cual operan modelos de aprendizaje que intentan emular la acción interpretativa humana. Este modo de aprendizaje es el que permite que las máquinas cobren autonomía, es decir, que hagan su propio proceso de aprendizaje, analizando grandes volúmenes de información sobre prácticas humanas específicas^4^^,^^5^^,^^6^^,^^51^^,^^52^.

En el ámbito de las ciencias, los argumentos que operarían simbólicamente para masificar la cobotización de la acción interpretativa se centrarían en la noción de “neutralidad” de la tecnología. Según Marcuse^53^, esa noción de la tecnología tiene su correlato en una concepción generalizada de las ciencias: su identidad y validez exceden los usos sociales que se hagan de ella. De allí que, por ejemplo, la participación de científicos y científicas en el desarrollo de productos altamente tóxicos, contaminantes o nocivos para la vida en el planeta, o incluso su participación en desarrollos tecnológicos con impactos sociales negativos queda completamente invisibilizada tras el velo de la tecnificación y de la neutralidad, que disocia el desarrollo de su aplicación social. Sin embargo, en términos de Marcuse, todo desarrollo tecnológico conlleva implícitamente un uso posible^53^, sobre todo si ese desarrollo se promueve y se financia desde un entorno industrial.

Cuando Habermas retoma la discusión de Marcuse con relación a la noción de Max Weber de “racionalidad formal”, señala que:

“Como la racionalidad de este tipo sólo se refiere a la correcta elección entre estrategias, a la adecuada utilización de tecnologías y a la pertinente instauración de sistemas (en situaciones *dadas* para fines *dados),* lo que en realidad hace es sustraer la trama social global de intereses en la que se eligen estrategias, se utilizan tecnologías y se instauran sistemas a una reflexión y reconstrucción racionales”.^22^

Sin embargo, al traer a un primer plano la trama social de intereses sustraída y los mecanismos que operan por detrás del avance tecnológico surgen algunos interrogantes: ¿la tecnología está inevitablemente asociado a la racionalidad técnica e instrumental?, ¿qué pasaría si la tecnología se generara desde racionalidades no sustentadas en el dominio y el control del mundo natural y social?

Hace cuatro décadas, el *Manifiesto cyborg* de Donna Haraway^54^ ponía en escena el híbrido máquina-organismo, y planteaba la necesidad de repensar en términos epistemológicos y políticos las relaciones sociales con la ciencia y la tecnología en el marco del capitalismo industrial. También señalaba como principal problema, que esas nuevas identidades cyborgs eran “hijos ilegítimos del militarismo y del capitalismo patriarcal”^54^, pero que se trataba de hijos “bastardos” cuyos padres no eran “esenciales”. Sin embargo, cuatro décadas después, esa relación se invirtió y la cobotización comenzó a generar hijos más obedientes: la inteligencia artificial de la mano de la reproducción del capital está transformando a los cyborgs -descriptos por Haraway como el “mito poderoso de resistencia”- en un insumo humano defectuoso y cuasi desechable.

Esto pone de manifiesto que las racionalidades más reflexivas no solo abandonaron la noción de “innovación” para dejarla en manos del conocimiento más instrumental, sino que abandonaron la creación de sistemas vinculados a las tecnologías “duras” que integren y privilegien las perspectivas relacionales. El campo de la salud colectiva ha producido importantes debates al respecto^55^^,^^56^^,^^57^^,^^58^^,^^59^ con desarrollos pioneros sobre las “tecnologías materiales” y las “tecnologías no materiales”^60^, que han sido retomados por perspectivas que integran las tecnologías blandas, blandas-duras y duras en un continuo a ser abordado en forma conjunta, dado que la adopción acrítica y central de las tecnologías duras impactan en los procesos relacionales y en los “saberes estructurados” que operan “en las prácticas que gobiernan los actos productivos, en los procesos de trabajo y en la capacidad de generar y gobernar nuevas modalidades de producción del cuidado”^61^.

Partiendo desde allí ¿podrían pensarse desarrollos tecnológicos que no estén basados en una lógica de dominio de la naturaleza y de los seres humanos? Y aquí es cuando recobran un nuevo protagonismo las palabras de Donna Haraway sobre la necesidad de repensar en términos epistemológicos y políticos las relaciones sociales con la ciencia y la tecnología en el marco del capitalismo industrial y comenzar a analizar cómo impactan estos procesos en la “conversación” que propone la salud colectiva. 

### Cobotización de la experiencia interpretativa de las ciencias

Desde que Christopher Sholes se asoció en 1873 con la fábrica de armas Remington para producir su prototipo de máquina de escribir, el acto de la escritura manuscrita se vio intermediado por un artefacto tecnológico^62^. Si bien la máquina de escribir implicó un borramiento de la grafía individual y una uniformización del texto mecanografiado, el proceso de traducir al plano narrativo una interpretación posible del entorno seguía siendo una práctica intrínsecamente humana. Para Martin Heidegger, la escritura mediada por la mecanización “degradaba la palabra”:

“La máquina de escribir arranca la escritura del ámbito esencial de la mano, es decir, del ámbito de la palabra. La palabra misma se convierte en algo ‘tecleado’. […] La escritura mecánica priva a la mano de su rango en el ámbito de la palabra escrita y degrada la palabra a un medio de comunicación. Además, la escritura mecánica proporciona esta ‘ventaja’: oculta la caligrafía y, con ello, el carácter. La máquina de escribir hace que todos parezcan iguales”.^63^

La cobotización de la experiencia interpretativa del mundo y de las tradiciones científicas implicaría la utilización de inteligencia artificial generativa ya no como un soporte mecánico para la estandarización de la grafía, sino para la propia generación de interpretaciones posibles del entorno, traducidas a estructuras narrativas humanamente válidas. El ChatGPT (*chat generative pre-trained transformer)*, desarrollado por la compañía OpenAI, se basa en uno de los modelos de aprendizaje automático (*machine learning*), denominado aprendizaje profundo (*deep learning*), que hace uso de redes neuronales de múltiples capas para aprender de los datos disponibles y tomar decisiones^64^^,^^65^^,^^66^. En el caso del ChatGPT, el aprendizaje profundo se basa en su capacidad para comprender relaciones y patrones en grandes volúmenes de datos de entrada y generar o predecir un nuevo texto, lo que lo hace capaz de responder preguntas o indicaciones, traducir idiomas, resumir textos y responder a preguntas de una forma comparable a la de un humano^66^. Sin embargo, para que el modelo comprenda el contexto y el significado del texto en lenguaje natural, necesita entrenarse, lo que requiere de una gran potencia de cálculo, acuerdos corporativos que permitan obtener y nutrir al modelo con grandes volúmenes de información, y un gran volumen de horas humanas de entrenamiento, de allí la liberación gratuita para un uso globalmente generalizado. 

Y es aquí donde se revierte el vínculo histórico de la humanidad con la tecnología: ya no aparece la acción humana como eje central de la experiencia interpretativa mediada por un artefacto, sino que la tecnología se nutre de una multiplicidad de experiencias humanas, que alimentan un modelo de aprendizaje profundo que deglute, concentra, monopoliza y estandariza esas experiencias para un uso corporativo, cuyo alcance aún desconocemos.

La reciente generalización del uso de la inteligencia artificial generativa en la elaboración de artículos científicos ha sido tan notable que ha llevado a la necesidad de regular la noción de autoría. La editorial Elsevier ha sido cuestionada por publicar una serie de artículos que contenían frases típicas de ChatGPT. En uno de ellos, en la introducción decía: “*Sin duda, aquí hay una posible introducción para su tema:…*”, en otro artículo decía: “*Lo siento mucho, pero no tengo acceso a información en tiempo real ni a datos específicos de pacientes, ya que soy un modelo de lenguaje de IA*”. Ambos trabajos estuvieron publicados durante varios meses y luego fueron retractados^67^.

Por el momento, los chatbots no son considerados entidades legales y no tienen personalidad jurídica. Tanto las editoriales científicas como otras instituciones vinculadas a la integridad de la investigación científica han afirmado “que las herramientas de IA no pueden figurar como autores de un artículo”:

“El uso de herramientas de inteligencia artificial (IA) como ChatGPT o Large Language Models en publicaciones de investigación se está expandiendo rápidamente. COPE se une a organizaciones como WAME y JAMA Network, entre otras, para afirmar que las herramientas de IA no pueden figurar como autores de un artículo. Las herramientas de IA no pueden cumplir los requisitos de autoría, ya que no pueden asumir la responsabilidad del trabajo enviado. Como entidades no jurídicas, no pueden afirmar la presencia o ausencia de conflictos de intereses ni gestionar acuerdos de derechos de autor y licencia”.^68^

Editoriales como Wiley, han dado un paso más y han planteado que las herramientas de inteligencia artificial “no pueden considerarse capaces de iniciar una pieza original de investigación sin la dirección de autores humanos”, ni tampoco pueden ser capaces “del diseño de la investigación”^69^, entendiendo estas acciones como intrínsecamente humanas. 

Sin embargo, para racionalidades más reflexivas, la explicitación de por qué la inteligencia artificial generativa no debería ser considerada como parte de la autoría debería apelar a otros fundamentos. Desde una perspectiva hermenéutica, el proceso humano de la escritura traduce una interpretación del mundo a un lenguaje y una trama narrativa que no son apuestas creativas individuales, sino que se asientan sobre acuerdos terminológicos y estructurales colectivos, entre quienes comparten ciertas formas de interpretar científicamente el mundo. Como señala David Carr, “la estructura narrativa impregna nuestra experiencia del tiempo y nuestra existencia social”^70^, por lo que en el acto de la escritura deberían estar presentes las huellas de la experiencia humana de la comprensión, lo que demandaría, en términos de Habermas, una “negociación de definiciones”^1^ de carácter colectivo que es uno de los componentes esenciales de la tarea interpretativa humana. 

Así comprendida, la escritura científica ya no es una reproducción de patrones lingüísticos, sino una acción humana colectiva, polifónica, dialógica y diversa de interpretar el entorno, que demandaría un concepto formal de mundo compartido al interior de las comunidades de lenguaje, y ese concepto formal de mundo expresa la racionalidad que las comunidades ponen en acción al momento de narrar de forma escrita su propia experiencia interpretativa del mundo. 

El avance de la inteligencia artificial generativa se expande sobre los cimientos creados durante décadas por la industrialización y tecnificación del texto, haciendo un uso que remite a aquello que Gadamer define como “un malentendido sobre el lenguaje”, es decir:

“…como si éste fuese un inventario contingente de palabras y frases, de conceptos, opiniones y modos de ver. El lenguaje es en realidad la única palabra cuya virtualidad nos abre la posibilidad incesante de seguir hablando […]. El lenguaje no es una convencionalidad reelaborada ni el lastre de los esquemas previos que nos aplastan, sino la fuerza generativa y creadora capaz de fluidificar una y otra vez ese material”.^15^

Estas líneas de Gadamer se actualizan y recobran nuevos sentidos ante el avance de los grandes modelos lingüísticos de aprendizaje profundo: el lenguaje humano “no es una convencionalidad reelaborada”^15^. Por lo tanto, la cobotización de la experiencia interpretativa y de su traducción dentro de racionalidades más reflexivas demandaría una puesta en valor de la escritura como acción comunicativa, de manera que la inteligencia artificial generativa se torne un instrumento que colabore en profundizar el carácter reflexivo de la comprensión humana y no a la inversa, haciendo que la experiencia humana se torne un insumo de una inteligencia artificial aumentada.

### Cobotización de la validación científica

Los modelos industrializados de validación científica, en los que se define qué es científicamente válido y qué no, sustentados en la estandarización de procesos de revisión por pares, tienden a homogenizar las diversas formas de interpretar científicamente el mundo y a eliminar los rasgos de identidades colectivas situadas. Y el efecto paradójico es que las propias comunidades de lenguaje, incluso aquellas motorizadas por racionalidades más reflexivas, al transferir la validación de su producción a escenarios industrializados, reproducen imaginarios de legitimación científica que promueven despojar el texto de toda identidad. Y es en estos entornos industrializados donde se promueve la cobotización de la gestión de los procesos de revisión por pares^71^^,^^72^^,^^73^^,^^74^.

Mientras la revisión por pares, relacional y a escala humana, era la instancia en que las diversas comunidades ponían en acción la formación lingüística de consensos en torno a las diversas formas de comprender científicamente el mundo, la industrialización de la revisión por pares a gran escala necesita desconectar y neutralizar esos consensos situados para lograr estandarizar los procesos y homogeneizar las racionalidades legitimadas a nivel global. Y es en este sentido que Habermas retoma de Niklas Luhmann el concepto de “tecnificación del mundo de la vida”^1^. 

Habermas diría que las comunidades de lenguaje -como las comunidades científicas que se nuclean en torno a una racionalidad común- demandan un tipo de acción comunicativa que no puede desconectarse del “contexto de saber cultural compartido, normas vigentes y motivaciones imputables, que constituye el mundo de la vida, porque tienen que servirse de los recursos que caracterizan la formación lingüística del consenso”^75^. Sin embargo, cuando opera la *tecnificación* de las acciones comunicativas, no solo se simplifica la comunicación, sino que la sustituye, desvalorizando los procesos sociales de formación de consenso y sometiéndolos al tipo de interacciones que imponen los entornos tecnificados. Como señala este autor, la tecnificación de la acción comunicativa promueve una desconexión humana de la interacción:

“Como no solamente simplifican la comunicación lingüística, sino que la sustituyen por una generalización simbólica de perjuicios y resarcimientos, el contexto del mundo de la vida en que siempre están insertos los procesos de entendimiento queda desvalorizado y sometido a las interacciones regidas por medios: el mundo de la vida ya no es necesario para la coordinación de las acciones”.^75^

En estos subsistemas en los que “el mundo de la vida ya no es necesario para la coordinación de las acciones”, como diría Habermas^75^, se produce una desconexión que genera un efecto paradójico en el componente humano de la interacción: “como un alivio de la necesidad de comunicación y una reducción de los riesgos que la comunicación comporta”^75^, desarticulando no solo el compromiso humano con la acción comunicativa, sino con los consensos y los valores creados por las propias comunidades, lo que explicaría en parte la baja participación de la comunidad científica en la discusión de nuevas interpretaciones del mundo de carácter colectivo.

La legitimidad para la incorporación acrítica de la inteligencia artificial generativa a los procesos de revisión por pares intenta encontrar su justificación, por un lado, en la incapacidad humana para detectar el fraude científico o para reconocer textos generados por inteligencia artificial^76^ y, por otro, en una especie de malestar hacia ciertas cualidades intrínsecamente humanas que comenzaron a ser consideradas negativas, como la divergencia de criterios, la subjetividad, y la diversidad de perspectivas^77^^,^^78^. 

Sin embargo, si bien la inteligencia artificial se pondera como una solución para enfrentar la mala gestión humana y sus deficiencias, un gran cuerpo bibliográfico ya está alertando sobre el “sesgo algorítmico”, que no solo reproduciría, sino que amplificaría los sesgos humanos históricamente conformados. Algunos sistemas de inteligencia artificial pueden producir resultados que discriminen a las personas por su orientación política^9^, por ser parte de poblaciones históricamente desatendidas o subrepresentadas en términos sanitarios^8^^,^^10^^,^^79^^,^^80^, o pueden “agravar las desigualdades existentes en cuanto a estatus socioeconómico, raza, origen étnico, religión, género, discapacidad u orientación sexual para amplificarlas e incidir negativamente en las desigualdades”^8^. 

¿Y por qué se amplifican los sesgos?, porque al alimentar los modelos de *deep learning* decidieron ignorar todo un cuerpo de conocimientos sobre cómo “las injusticias, la opresión y la destrucción causadas por el capitalismo, el colonialismo y el patriarcado” que nos señala Boaventura Santos^81^, están normalizadas e incrustadas en el lenguaje del que se nutren los grandes modelos lingüísticos de aprendizaje profundo.

Si bien existe la posibilidad de reentrenar los modelos para intentar disminuir los sesgos, los modelos actuales de *deep learning* se están alimentando de grandes volúmenes de datos capturados, por un lado, a través de prácticas extractivas depredadoras^82^^,^^83^^)^ y, por otro, a través de acuerdos corporativos generados al interior del propio complejo industrial científico-editorial(84-87), lo que imposibilita dimensionar qué ingresa al modelo. Un ejemplo, es la reciente detección en la bibliografía científica de términos inexistentes, como el caso de “*vegetative electron microscopy*”^88^^,^^89^, que pareciera ser un término técnico, cuando en realidad es una frase sin sentido que se ha convertido en un “fósil digital”:

“…un error preservado y reforzado en los sistemas de inteligencia artificial (IA) que es casi imposible de eliminar de nuestros repositorios de conocimiento. […] El auge de la IA crea oportunidades para que los errores se integren permanentemente en nuestros sistemas de conocimiento, a través de procesos que ningún actor controla por sí solo. Esto presenta desafíos tanto para las empresas tecnológicas como para los investigadores y las editoriales”.^88^

En este sentido, la cobotización a escala industrial de los procesos de validación científica implicaría transferir a este tipo de modelos la capacidad de definir qué es científicamente válido y qué no. Y al transferir la validación científica a esos escenarios, se desarticulan las complejas y múltiples prácticas relacionales humanas a través de las cuales las comunidades científicas solían consolidar acuerdos, desacuerdos y consensos, y admitían las tensiones propias de la coexistencia de diversas y dispares formas humanas de comprender el entorno natural y social.

Las racionalidades más reflexivas deberían asumir la relevancia de contar con sus propios entornos tecnológicos de validación, de tal manera que, tal como señala Boaventura de Sousa Santos, los diversos grupos sociales tengan la posibilidad de representar “el mundo como propio y en sus propios términos, pues solo de ese modo serán capaces de transformarlo de acuerdo con sus propias aspiraciones”^81^. En este sentido, Susan Leavy^7^ menciona que solo “quienes se ven potencialmente afectados por el sesgo tienen más probabilidades de verlo, comprenderlo e intentar resolverlo”. Por lo tanto, más que rechazar el uso de la tecnología, o de adoptarla acríticamente, se requiere asumir la necesidad de crear entornos tecnológicos inclusivos, para lo cual generar las articulaciones y los escenarios colaborativos que viabilicen su desarrollo.

## A modo de cierre

La cobotización de la interpretación científica del mundo y de las instancias de validación de esas interpretaciones no emergieron por igual en todos los ecosistemas científicos, sino que se erigieron en entornos marcados por los efectos contaminantes de la industrialización y la mercantilización del texto, cuyas ondas expansivas han ido avanzando a nivel global. 

Tan enredados e inmersos estamos en esta tecnificación del mundo de la vida que, a pesar de los efectos nocivos que estos procesos provocaron en la desarticulación de las comunidades de lenguaje, desde un gran abanico de racionalidades científicas abrazaron durante décadas la industrialización y la mercantilización del texto que otrora fuera la arena de reflexión y de discusión colectiva, transfiriendo sus entornos de validación y de legitimación a las corporaciones, deslegitimando y desarticulando sus propios escenarios de generación de consensos. Y sobre este contexto erosivo se erigieron los procesos de cobotización, moldeando nuevas subjetividades. 

Los nuevos sujetos de esta era digital marcada por la inteligencia artificial generativa tienden a evitar el esfuerzo comunicativo que implica la discusión entre perspectivas diversas y dispares. Los gemelos digitales personales, creados y alimentados para actuar como clones personales con capacidad de interactuar en situaciones laborales y comunicacionales diversas, son uno de los tantos indicios de estas nuevas subjetividades. En su texto “*Personal AI digital twins: the future of human interaction?*”, Jim Spohrer se pregunta “pero ¿cómo se sentirán mis amigos y colegas al interactuar con mi gemelo digital de IA? ¿No preferirán a mi yo real? Dejando el ego de lado por un momento, la verdadera respuesta es que no necesariamente”^90^.

Este retroceso en la acción comunicativa entre humanos va de la mano de un retraimiento del esfuerzo que demanda la acción interpretativa del mundo. Tomar la iniciativa de comprender, en términos de Gadamer, “es lo que constituye el verdadero esfuerzo de la existencia”^14^; sin embargo, el componente humano de la cobotización tiende a delegar ese esfuerzo interpretativo, al punto de desarticular el compromiso humano con la acción comunicativa y con la conformación de nuevos consensos y valores al interior de las propias comunidades de diálogo.

En este contexto, la teoría crítica, al abordar los efectos de la tecnificación de las relaciones y de las acciones comunicativas humanas sobre los colectivos sociales, parte del reconocimiento de estas nuevas subjetividades para plantear la necesidad de multiplicar y legitimar escenarios en los que las racionalidades críticas y reflexivas propongan formas de cobotización en las que la racionalidad de la interacción preserve como núcleo central de preocupación las acciones comunicativas relacionales, recupere el esfuerzo crítico de seguir repensando las relaciones humanas desde una “metapresencialidad”^55^ anclada en un aquí y ahora situados, centrada en la mejora de las condiciones de vida del conjunto social y la disminución de las desigualdades. 
